# Digital Signal Processing and Control for the Study of Gene Networks

**DOI:** 10.1038/srep24733

**Published:** 2016-04-22

**Authors:** Yong-Jun Shin

**Affiliations:** 1 Biomedical Engineering Department, University of Connecticut, Storrs, CT 06269, USA

## Abstract

Thanks to the digital revolution, digital signal processing and control has been widely used in many areas of science and engineering today. It provides practical and powerful tools to model, simulate, analyze, design, measure, and control complex and dynamic systems such as robots and aircrafts. Gene networks are also complex dynamic systems which can be studied via digital signal processing and control. Unlike conventional computational methods, this approach is capable of not only modeling but also controlling gene networks since the experimental environment is mostly digital today. The overall aim of this article is to introduce digital signal processing and control as a useful tool for the study of gene networks.

Digital signal processing and control engineering has been widely used in many areas of science and engineering today^1^,^2^. It enables our “digital society” and its applications are vast. This popularity is a result of the significant advances in digital computer technology. Complex signal processing and control tasks, which are usually too difficult and/or too expensive to be performed by analog systems, can be performed by less expensive and often more reliable digital computers. Furthermore, digital signal processing and control algorithms provide a greater degree of flexibility as they are programmable. In this context, there has been an explosive growth in digital signal processing and control theory and applications over the past decades. In this article, it is proposed the digital approach can be useful for the study of gene networks. Unlike conventional modeling approaches such as Boolean networks, Bayesian networks, Petri nets, ordinary differential equations, and stochastic simulation algorithms (reviewed in^3^), digital signal processing and control can be used not only to model, simulate, and analyze gene networks but also to interact with them in real time as experimental data are mostly digital today. Analog or continuous experimental data are sampled or discretized at discrete time points (analog-to-digital conversion) and processed to generate digital control signals, which are transformed into analog signals through digital-to-analog conversion (Fig. 1A). While calculus-based differential equations dominate in continuous domain, discrete-time difference equations, which require only addition and multiplication, become useful in digital domain^1^,^2^. Difference equations have been extremely powerful in computational science and engineering due to this simplicity^4^. They are typically generated by discretizing continuous differential equations using various approaches such as the Euler and Runge-Kutta methods^5^. This approach is based on the assumption that continuous models better reflect reality and difference equations can be used to “approximate” those continuous models. Approximation or discretization error can be decreased by increasing the discretization resolution or the sampling frequency. In this article, an alternative approach is proposed as gene network dynamics are inherently discrete in nature. For example, there is no such thing as 1.02437 protein molecule. The amount of protein molecules present in cells is always a discrete integer number. In addition, the production of each protein molecule requires a discrete amount of time. In this context, it is proposed that difference equations can be used to model gene network dynamics, which approximate not continuous differential equations but real systems. This approach is not only simpler but also more accessible to students and researchers not familiar with differential equations since the mathematics is simple to understand. Similar to the first approach, the approximation or discretization error can be decreased by increasing the discretization resolution or the sampling frequency.

Proteins are the worker molecules of biological systems. They perform virtually every activity within living organisms, including metabolism, cell division, apoptosis (programmed cell death), cell-cell interaction, etc. Therefore, it is not surprising that the biological information that each gene encodes is mainly for producing a specific type of protein. However, only knowing how a gene modulates its protein production is often insufficient to fully capture protein dynamics. A gene can activate or suppress the activity of other genes. This coupling or interaction of genes is called gene networks and any protein dynamics should to be understood in this context. It was suggested that gene networks are made of a small set of recurring modules called network motifs^6^,^7^.

Network motifs include simple two-gene network, autoregulation, feedforward loop, and feedback loop. The complexity of gene networks originates not only from various interconnection patterns or the number of genes involved but also from their adaptive and robust features^8^,^9^,^10^,^11^,^12^,^13^,^14^. Although understanding these features is important to study the controllability of complex gene networks and to eventually control them^15^, a mathematical framework is not yet well-established. It is an on-going research topic not only for biology but also for other related fields, such as adaptive sensor networks and swarm robotics, which involve complex network dynamics^9^,^16^,^17^,^18^.

The overall aim of this article is to introduce digital signal processing and control as a useful tool for the study of gene networks. Since digital signal processing and control, not to mention gene networks, is a very broad field that encompasses a wide range of topics, it is impossible to systematically probe every topic in depth. Therefore, a sequence of topics and examples are presented in a way that motivates the readers to pursue them further. Every attempt was made to make the materials accessible not only to engineers, mathematicians, or physicists but also to life scientists, introducing the basics of both digital signal processing and control and gene networks.

## Results

### Simple two-gene network

**Figure 1:**
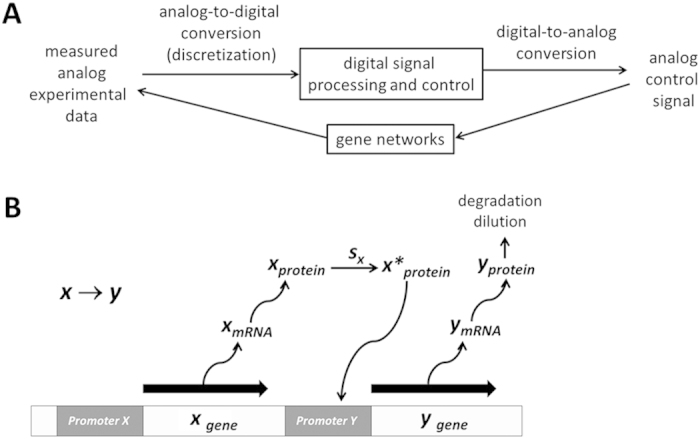
(**A**) Digital signal processing and control. Analog or continuous experimental data measured are sampled or discretized at discrete time points (analog-digital conversion) and processed to generate digital control signals, which are transformed into analog signals through digital-to-analog conversion. (**B**) Simple two-gene network. First, *x*_*gene*_ is transcribed into *x*_*mRNA*_, which is then translated into *x*_*protein*_. In the presence of signal *s*_*x*_, *x*_*protein*_ transforms into its active form x*_protein_ and binds to the promoter of *y*_*gene*_, transcribing *y*_*gene*_ into *y*_*mRNA*_. As *y*_*mRNA*_ is translated, *y*_*protein*_ is produced. The effect of *y*_*protein*_ production by *x*_*protein*_ can be counteracted by two processes that decrease *y*_*protein*_ concentration: degradation (protein destruction) and dilution (concentration reduction due to increased cell volume).

Modeling simple two-gene network as a difference equation becomes important since it can serve as the basic building block for constructing more complex gene networks^11^. One gene (*y*_*gene*_) can be activated by another gene (*x*_*gene*_), as indicated by the notation *x* → *y* in Fig. 1B. This simple notation, however, involves multiple steps. First, *x*_*gene*_ is transcribed into *x*_*mRNA*_, which is then translated into *x*_*protein*_. In the presence of signal *s*_*x*_, *x*_*protein*_ transforms into its active form x^*^_protein_ and binds to the promoter of *y*_*gene*_, transcribing *y*_*gene*_ into *y*_*mRNA*_. As *y*_*mRNA*_ is translated, *y*_*protein*_ is produced. Since protein activity is generally governed by concentration, we will express *x*_*protein*_ and *y*_*protein*_ in terms of concentration. The effect of *y*_*protein*_ production by *x*_*protein*_ can be counteracted by two processes that decrease *y*_*protein*_ concentration: degradation (protein destruction) and dilution (concentration reduction due to increased cell volume).

First, let us assume that *y*_*protein*_ is only degraded or diluted. There is no *y*_*protein*_ production by x^*^_protein_. This can be modeled using a difference equation:


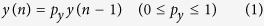


where n is the discrete time index (integer) and *y*(n) is the concentration of *y*_*protein*_ at time n. Having a sufficiently small time interval between measurements is important to capture desired dynamics in detail (Nyquist sampling theorem^1^). In case we measure *y*_*protein*_ and x^*^_protein_every minute, the time difference between *y*(1) and *y*(2) is 1 minute. *p*_*y*_ can be any value between 0 and 1. It cannot be greater than 1, which makes *y*_*protein*_ increase, or less than 0, which makes *y*_*protein*_ negative. In case *y*(1) = 10 μM and *p*_*y*_ = 0.9:


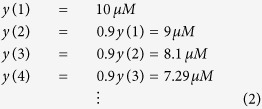


which shows *y*_*protein*_ is indeed decreasing over time. The simulation code for Eq. 1 can be found online (see learnsysbio.net **Module 1**^19^). If we lower *p*_*y*_ from 0.9 to 0.1:


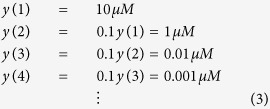


which indicates *y*_*protein*_ is more rapidly decreasing. In other words, a smaller *p*_*y*_ value corresponds to greater degradation or dilution.

Next, let us add a new term *p*_*xy *_*x*(n − 1) to Eq. 1 to model *y*_*protein*_ production by x^*^_protein_:


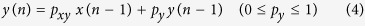


where n is the discrete time index (integer) and *y*(n) is the concentration of *y*_*protein*_ at time n and *x*(n) is the concentration of x*_protein_at time n. Note the value *y*(n) at time n now depends on the previous values *y*(n−1) and *x*(n −1) at time n - 1. The parameter *p*_*xy*_shows how strongly x*_protein_ activating *y*_*gene*_. *p*_*xy*_can be affected by many factors, including the presence of signal *s*_*x*_, transcription factor/promoter binding strength, etc. It cannot be indefinitely large since *y*_*protein*_ production is restricted by the finite amount of available protein production machineries such as ribosomes.

An example with constant *x*(n) values (=10 μM), *y*(1) = 0 μM, *p*_*y*_ = 0.9, and *p*_*xy*_ = 0.2, can be shown as:


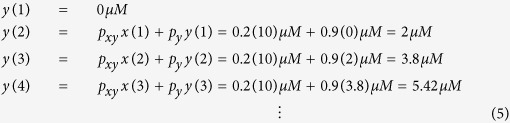


**Figure 2:**
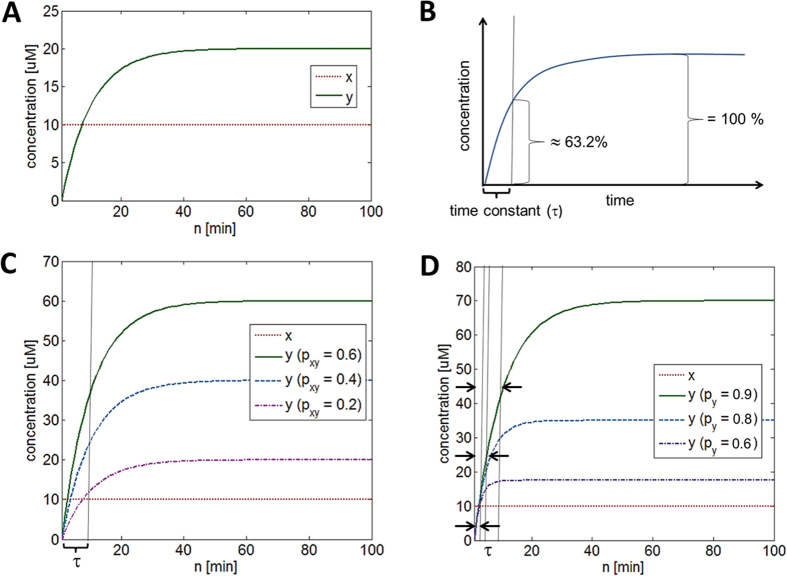
(**A**) The simulation result of Eq. 4. *y*_*protein*_ reaches a constant or steady-state level as time approaches infinity while *y*_*protein*_ production and degradation and/or dilution are simultaneously occurring. (**B**) The time constant τ (tau) represents the time it takes *y*_*protein*_ to reach approximately 63.2% of the steady-state level. It is also called the response time, which can be used to evaluate how fast *y*_*protein*_ changes or responds to the input signals. (**C**) The effect of different *p*_*xy*_ values on *y*_*protein*_. As *p*_*xy*_ increases, the steady-state level also increases because x*_protein_is increasingly activating *y*_*gene*_. However, the time constant τ does not change, indicating that *p*_*xy*_, which governs *y*_*protein*_production, does not affect how fast *y*_*protein*_ reaches its steady-state level. (**D**) The effect of different *p*_*y*_ values on *y*_*protein*_. As *p*_*y*_ decreases (degradation increases), the steady-state level decreases because *y*_*protein*_ is degraded or diluted more. Note the time constant τ also decreases, which means *y*_*protein*_ reaches its steady-state level faster as *p*_*y*_ decreases.

The simulation code for Eq. 5 can be found online (see learnsysbio.net **Module 2**^19^) and the simulation result is shown in Fig. 2A. From Eq. 5, following expressions can be derived:


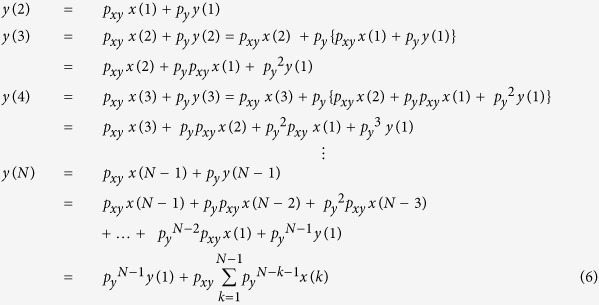


Eq. 6 shows that as time approaches infinity (N ≈ ∞) *y*_*protein*_ reaches a steady-state level given that *p*_*y*_ < 1 and *p*_*xy*_and *x*_*protein*_ values are not indefinitely large. This steady-state level is achieved while *y*_*protein*_ production and degradation and/or dilution are simultaneously occurring. The time constant τ (tau) represents the time it takes *y*_*protein*_ to reach 1 −1/e or approximately 63.2% of the steady-state level (Fig. 2B). It is also called the response time, which can be used to evaluate how fast *y*_*protein*_ changes or responds to the input signals.

### Protein production and degradation exhibit different dynamics

**Figure 3:**
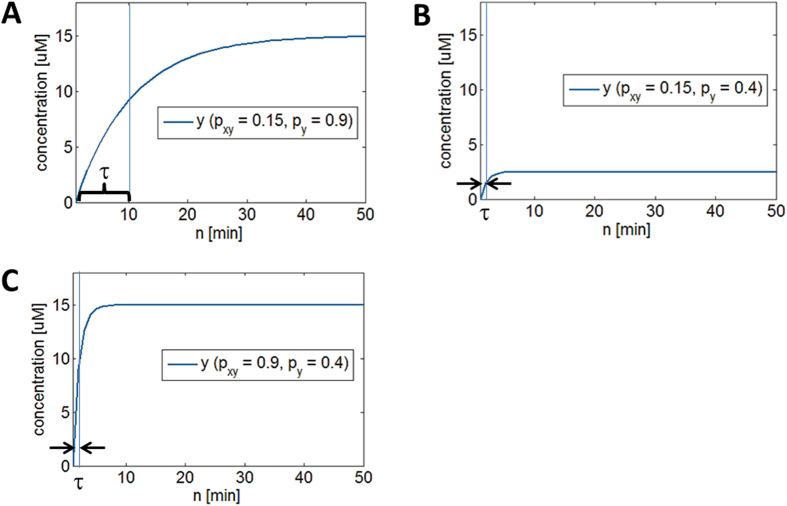
Achieving target steady-state level with desired response time. (**A**) A target steady-state level (approximately 15 μM) is achieved with *p*_*xy*_ = 0.15 and *p*_*y*_ = 0.9. The response time is about 10 minutes. (**B**) Decreasing *p*_*y*_from 0.9 to 0.4 (increasing protein degradation) reduces the response time to approximately 2 minutes, which is desired. However, the steady-state level is also substantially reduced as a side effect. (**C**) The target steady-state level of 15 μM with the response time of 2 minutes can be achieved by increasing *p*_*xy*_ from 0.15 to 0.9 while maintaining low *p*_*y*_ (high protein degradation).

Figure 2C shows the effect of different *p*_*xy*_ values on *y*_*protein*_. As *p*_*xy*_ increases, the steady-state level also increases because x*_protein_is increasingly activating *y*_*gene*_. However, the time constant τ does not change, indicating that *p*_*xy*_, which governs *y*_*protein*_production, does not affect how fast *y*_*protein*_ reaches its steady-state level (see learnsysbio.net **Module 3**^19^). Figure 2D shows the effect of different *p*_*y*_ values on *y*_*protein*_. As *p*_*y*_ decreases (degradation increases), the steady-state level decreases because *y*_*protein*_ is degraded or diluted more. Note the time constant τ also diminishes, which means *y*_*protein*_ reaches its steady-state level faster as *p*_*y*_ decreases (see learnsysbio.net **Module 4**^19^). In summary, protein production affects only steady-state level, while protein degradation can affect both steady-state level and response time. This difference can be used to achieve target steady-state level with desired response time as shown in Fig. 3. Figure 3A shows a particular target steady-state level (approximately 15 μM) can be achieved with *p*_*xy*_ = 0.15 and *p*_*y*_ = 0.9. The response time is about 10 minutes. Decreasing *p*_*y*_from 0.9 to 0.4 (increasing protein degradation) reduces the response time to approximately 2 minutes, which is desired (Fig. 3B). However, the steady-state level is also substantially reduced as a side effect. How can we achieve the target steady-state level of 15 μM with the response time of 2 minutes ? Figure 3C shows this can be done by increasing *p*_*xy*_ from 0.15 (Fig. 3A) to 0.9 while maintaining low *p*_*y*_ (high protein degradation). In other words, we can achieve the target steady-state level with desired response time by co-modulating protein production (*p*_*xy*_) and degradation (*p*_*y*_).

**Figure 4:**
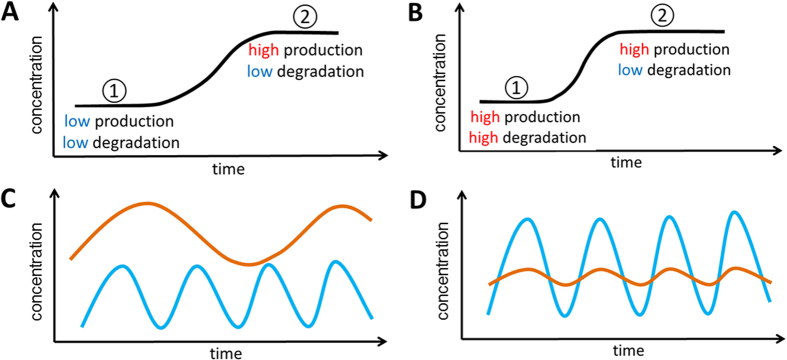
Co-modulating protein production and degradation (and/or dilution). (**A**) By increasing protein production from low to high, a transition can occur from Level 1 to Level 2. Note degradation is low in both Level 1 and Level 2. (**B**) Level 1 can also be achieved with high production and high degradation. In this case, lowering degradation enables a faster transition from Level 1 to Level 2. (**C**) Protein pulses with high (blue) and low (brown) frequencies. High-frequency protein pulses (blue) show short pulse duration while low-frequency protein pulses (brown) show longer pulse duration. (**D**) Protein pulses with high (blue) and low (brown) amplitudes.

Protein production and degradation are also “asymmetric”. Protein production is a slow process compared to protein degradation and this asymmetry can be exploited to generate various protein dynamics. An example is illustrated in Fig. 4. Let us assume cells want to change protein level from Level 1 to Level 2 by increasing its production as shown in Fig. 4A. Since protein production is a slow process, increasing its rate is also slow. Note degradation is low in both Level 1 and Level 2. Cells can also achieve Level 1 with high production and high degradation as shown in Fig. 4B. In this case, Level 2 can be reached by lowering degradation. Since production is already at high speed, the transition from Level 1 and Level 2 occurs faster. Using this co-modulation in various ways, cells may generate pulses with desired frequency, amplitude, and duration (Fig. 4C,D), which has biological significance in DNA repair, tumorigenesis, embryonic development, etc.^20^,^21^,^22^.

### Robustness of steady-state level

**Figure 5:**
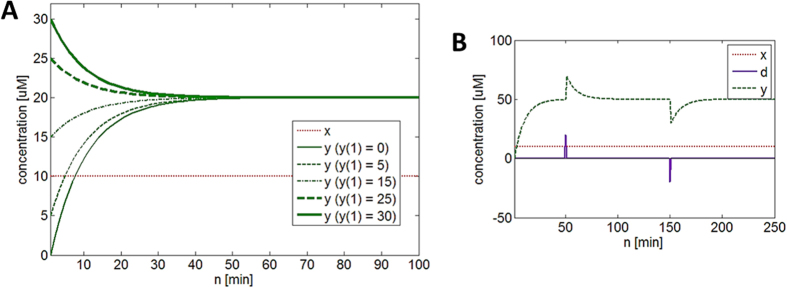
(**A**) Different initial values do not affect the steady-state level. (**B**) Steady-state level “robustly” maintains and restores its value in the presence of disturbance *d*.

The first term of Eq. 6, which depends on *y*(1) or the initial value of *y*_*protein*_, becomes zero as time goes to infinity, indicating that the steady-state level does not depend on the initial value. For example, if we change *y*(1) from 0 to 5 μM, the steady-state level will not be affected (see Fig. 5A and learnsysbio.net **Module 5**^19^). Furthermore, steady-state level can be robustly maintained while automatically restoring its value in the presence of disturbance (see Fig. 5B and learnsysbio.net **Module 6**^19^). The *y*_*protein*_ dynamics defined by Eq. 4 enables this robustness of steady-state level.

### Matrix representation of simple two-gene network

Matrix computation is widely used in computational science and engineering^23^ and representing gene network dynamics using matrices and vectors can be useful. From Eq. 4, following expressions can be derived:


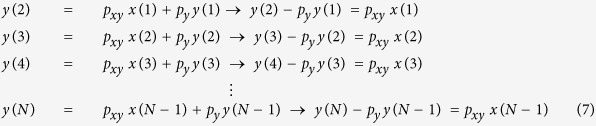


These expressions can be compactly represented using matrices and vectors:


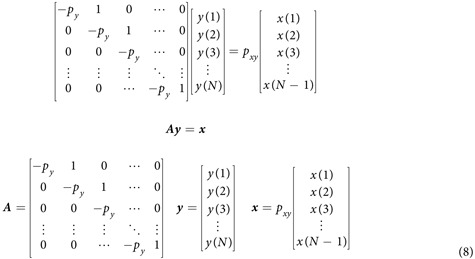


Given ***A*** and ***x*** we can find ***y***, which becomes solving a system of linear equations.

### Basal and saturated production

*y*_*protein*_ can be produced without being activated by other genes, which is called basal production. This production does not depend on x*_protein_and can be denoted as *b*_*0*_:


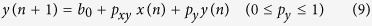


When simulating gene networks, basal production can be added to the model to prevent protein levels from becoming negative. *y*_*protein*_ cannot indefinitely increase as x*_protein_increases because *y*_*protein*_ production eventually becomes saturated due to limited availability of protein synthesis resources (e.g., ribosomes). This can be modeled as:


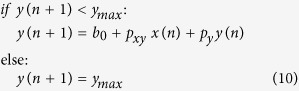


where *y*_*max*_is the maximum level *y*_*protein*_ can be. We will use Eq. 4, which does not consider basal and saturated production, for simplicity in this article.

### Stochastic modeling: extrinsic and intrinsic noise

**Figure 6:**
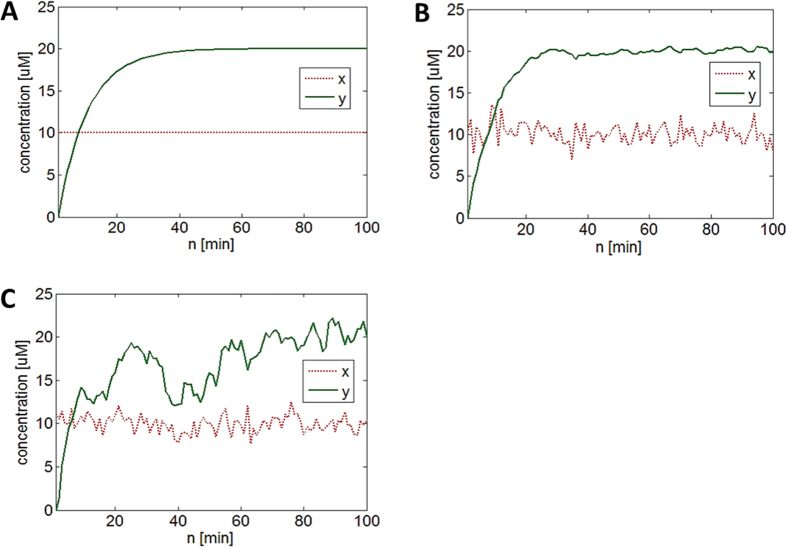
Extrinsic and intrinsic noise. (**A**) There is no noise affecting *x*_*protein*_ and *y*_*protein*_ levels. (**B**) Extrinsic or global noise obeying a normal distribution (mean: 0, standard deviation: 1) is added to *x*_*protein*_ only. Although no noise is added to *y*_*protein*_, fluctuations in *y*_*protein*_ levels are observed, which is intrinsic noise caused by extrinsic noise propagated from *x*_*protein*_ to *y*_*protein*_. (**C**) Extrinsic noise is added to *y*_*protein*_.

Biological processes including gene expression are inherently stochastic constantly affected by noise^24^,^25^,^26^. Different types of noise influencing gene expression include extrinsic and intrinsic noise^24^. Extrinsic noise is “global” and universally affects the expression of all genes in a given cell. It is generated by factors such as variations in the number of RNA polymerase, ribosome, etc. In contrast, intrinsic noise is “local” and caused by noise propagated from upstream genes to downstream genes. It is generated by the randomness inherent in gene expression and has been used to analyze gene regulatory links^27^. In our simple two-gene network model, *x*_*gene*_ (upstream gene) activates *y*_*gene*_ (downstream gene), which means extrinsic noise affecting *x*_*gene*_ gene expression can be propagated to create intrinsic noise in *y*_*gene*_ gene expression. This is demonstrated in Fig. 6. Initially, there is no noise affecting *x*_*protein*_ and *y*_*protein*_ levels (Fig. 6A). Extrinsic noise obeying the normal (Gaussian) distribution (mean: 0, standard deviation: 1) is only added to *x*_*protein*_ (see Fig. 6B and learnsysbio.net **Module 7**^19^). Although no noise is added to *y*_*protein*_, fluctuations in *y*_*protein*_ levels (intrinsic noise) are observed, which is caused by extrinsic noise propagated from *x*_*protein*_ to *y*_*protein*_. Interestingly, *y*_*protein*_ fluctuations are much less than *x*_*protein*_ fluctuations because our simple two-gene network is a low-pass filter, which removes or reduces high-frequency noise signals. This will be further discussed later. When extrinsic noise is added to *y*_*protein*_ as well, *y*_*protein*_ fluctuations look similar to *x*_*protein*_ fluctuations (see Fig. 6C and learnsysbio.net **Module 8**^19^).

### Predicting *y*_*protein*_ levels

When measured *x*_*protein*_ and *y*_*protein*_ data (e.g., Fig. 6C) are available, the parameter values *p*_*xy*_ and *p*_*y*_ of Eq. 4 can be estimated, which can be used for predicting unknown *y*_*protein*_ level. There are mainly two models we can use for this prediction: 1) time-invariant models with fixed parameter values and 2) time-variant models with parameter values changing adaptively over time. Gene networks are often treated as time-invariant by computational models but it was previously demonstrated that adaptive time-variant models approximate experimental measurements more accurately than time-invariant models, while reducing modeling complexity and representing gene network dynamics more realistically^10^. Before proceeding any further, Eq. 4 can be modified into a more general form:


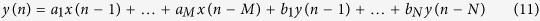


where *y*(n) at time n depends not only on immediate previous values *x*(n −1) and *y*(n −1) but also on other older *x* and *y* values (e.g., *x*(n −3) and *y*(n −5)). There are *M* previous *x* terms and *N* previous *y* terms determining the current value of *y*(n). The parameters *a*_*1*_, …, *a*_*M*_ and *b*_*1*_, …, *b*_*N*_ show how each previous *x* and *y* term affects *y*(n). In case the values of these parameters are fixed, Eq. 11 represents a time-invariant model. In contrast, an adaptive time-variant model allows them to change over time. Let us first examine a time-invariant model with fixed parameter values. The parameters or weights can be compactly represented by a single column vector ***w***:


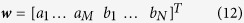


where superscript *T* stands for transpose, which changes a row vector into a column vector. The data vector ***d*** can be defined as:


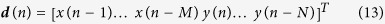


For example, in case we define our model as:


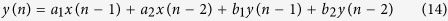


***w*** and ***d*** can be expressed as:


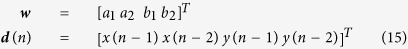


Using Eqs 12 and 13, Eq. 11 can be compactly expressed as:


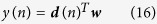


Assuming ***w***_*opt*_ is the parameter vector computed in an optimal way:


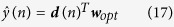


where and 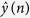 is the estimated value of *y*(*n*), which can be measured experimentally. The difference or error between *y*(*n*) and 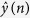 can be denoted as *e*(*n*):


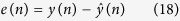


One approach for finding ***w***_*opt*_ is using the Least Squares (LS) estimation method such that ∑*e*(*n*)^2^ is minimized^4^,^10^:


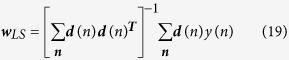


where ***w***_*LS*_ is ***w***_*opt*_ computed by the method. Applying Eq. 19 to our previous example model (Equations 14 and 15), we get:


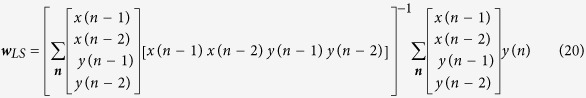


Once ***w***_*LS*_ is found, 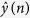 can be predicted using Eq. 17:


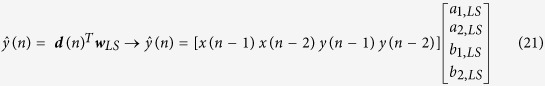


**Figure 7:**
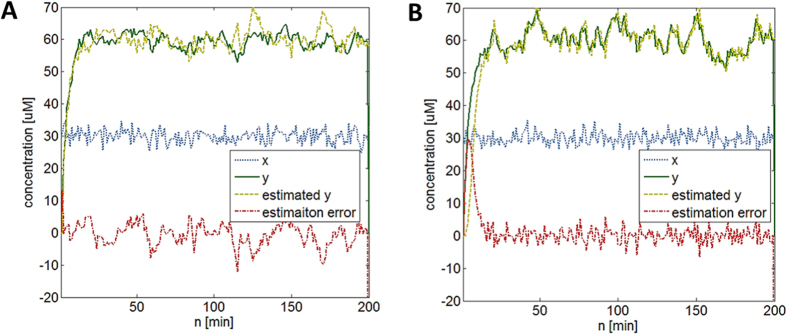
(**A**) *y*_*protein*_ level prediction using the Least Squares (LS) method. (**B**) *y*_*protein*_ level prediction using the Least Mean Squares (LMS) method. The estimation error is large in the beginning but soon substantially reduced as the filter “learns” adaptively over time.

Figure 7A shows an example of *y*_*protein*_ prediction using the least squares approach. Simulated data similar to Fig. 6C are used. As discussed previously by the author^10^, the estimation performance using this approach is generally poor due to highly “time-varying” nature of gene expression even if we increase the model complexity by adding more terms to Eq. 14.

### *y*_*protein*_ level prediction using the Wiener filter

There is another approach for *y*_*protein*_ prediction using a time-invariant model called the Wiener filter^28^. The Wiener filter computes ***w***_*opt*_ that minimizes the expected value of *e*(*n*)^2^. The concepts of random variable, discrete-time random process, and correlation will be briefly reviewed before they are discussed^29^. Let us assume that we measure *y*[*n*] using fluorescent reporter technology^30^. *y*(82), the *y*_*protein*_ level at time 82 (n = 82), is called a random variable whose value can randomly change in each experiment. A collection of these random variables (e.g., *y*(1), *y*(2), *y*(3), …) at discrete time points is called a discrete-time random process. The expected value of random variable *y*(n) can be defined as:


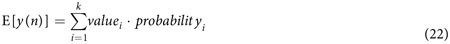


where *k* is the number of values *y*(n) can take. In theory, we need an infinite number of experiment results to find the exact expected value of a random variable. The autocovariance function, which shows how two random variables that belong to the same (“auto”) random process “co-vary”, can be defined as:


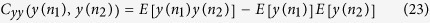


where n_1_ and n_2_ can be any time point from the identical discrete-time random process *y*(n). The first term of Eq. 23 is the autocorrelation function:


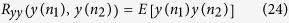


The cross-covariance function, which shows how two random variables that belong to two different (“cross”) random processes “co-vary”, can be defined as:


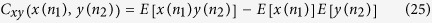


where *x* is a discrete-time random process formed by random variables x(1), x(2), x(3), …. The first term of Eq. 25 represents the cross-correlation function:


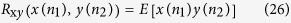


The Weiner filter can be used to estimate the parameters shown in Eq. 12 using autocorrelation and cross-correlation:


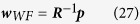


where the correlation matrix ***R*** is *E*[***d***(*n*)***d***(*n*)^*T*^] and the correlation vector ***p*** is 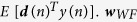 is ***w***_*opt*_ computed by the Wiener filter. Once ***w***_*WF*_ is found, it can be used to predict 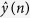 (Eq. 17):


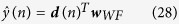


Although the Weiner filter can perform better than the Least Squares method (Eq. 19), it is based on the assumption that *x* and *y* are wide-sense stationary random processes and may not perform well if *x* and *y* are non-stationary random processes^28^,^29^. Furthermore, finding expected values requires a substantial number of prior experiments to be performed, which is often not possible. Most of all, the approach assumes that the parameter values to be estimated are fixed and do not change over time. However, biological systems are highly dynamic and it is possible that those values will constantly change, demanding tracking of such fluctuating parameter values. Adaptive filters address these issues.

### *y*_*protein*_ level prediction using adaptive filters

Adaptive filters iteratively update ***w***_*opt*_(*n*) that attempts to minimize *e*(*n*)^2^. The Least Means Squares (LMS) filter achieves this using the stochastic gradient descent method^31^:


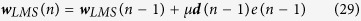


where ***w***_*LMS*_(*n*) is ***w***_*opt*_(*n*) computed using LMS at time n and *μ* is the step size, which can be tuned to optimize performance. Let us use LMS to estimate the time-varying parameter values of simple two-gene network model shown in Eq. 4 (a_1_ = *p*_*xy*_ and *b*_1_ *=* *p*_*y*_):


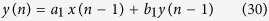



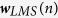
 and ***d***(*n*) can be defined as:


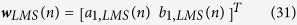



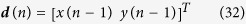


Note ***w***_*LMS*_(*n*) is not fixed and can constantly change over time. Using Eqs 31 and 32, 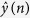 can be compactly expressed as:


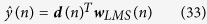


The difference or error between measured 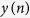 and estimated 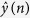 can be denoted as 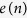:


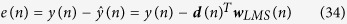


Using Eqs 31 and 32, Eq. 29 can be re-expressed as:


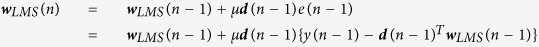



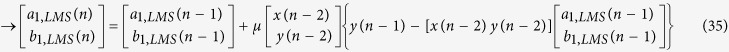


Since 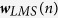 is known, 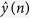 can be computed:


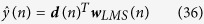


Once measured *y*(*n*) is available, ***w***_*LMS*_(*n* + 1) can be computed, which is then used to estimate 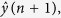 and these steps are repeated. An example of LMS-enabled *y*_*protein*_ level estimation is shown in Fig. 7B (also see learnsysbio.net **Module 9**^19^). Note the estimation error *e*(n) is large in the beginning but soon substantially reduced as the filter “learns” adaptively over time. Taking time variation into consideration allows low-order simple models (e.g., Eq. 30, Fig. 7B) to outperform more complex higher-order, time-invariant models (e.g., Eq. 14, Fig. 7A)^10^. Although adaptive filtering is introduced as an estimation technique for better fitting a model to data, the same approach can be used to elucidate the adaptive behavior of biological systems^9^,^13^,^14^. Another possible extension of this work is to use adaptive filters and various forms of control mechanisms, such as adaptive control methods, for identifying and controlling the stochastic dynamics of gene networks in real time as described later.

### Simple two-gene network is a low-pass filter

**Figure 8:**
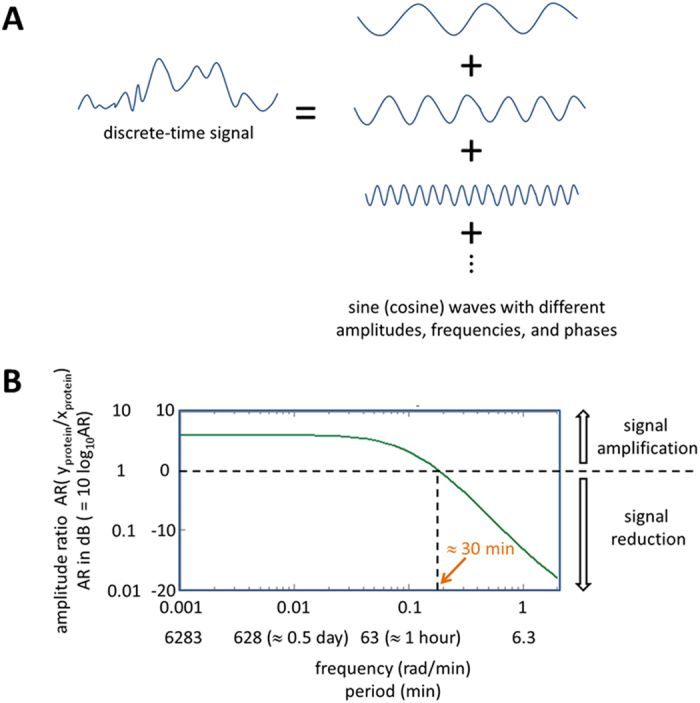
(**A**) Any discrete-time signal (time-domain) can be decomposed into a collection of sine or cosine waves with different amplitudes, frequencies, and phases (frequency domain). (**B**) The frequency response of *H*(*z*) can be illustrated using Bode plot. The effect of extrinsic noise signals added to *x*_*protein*_ levels with cycle period shorter than 30 min (high-frequency noise) will be reduced in *y*_*protein*_ expression, indicating Eq. 4 is a low-pass filter that passes low-frequency *x*_*protein*_ signals while attenuating high-frequency *x*_*protein*_ signals.

Any discrete-time signal (time-domain) can be decomposed into a collection of sine or cosine waves with different amplitudes, frequencies, and phases (frequency domain) (Fig. 8A). Using the z-transform, Eq. 4 can be converted into its frequency domain representation^2^:


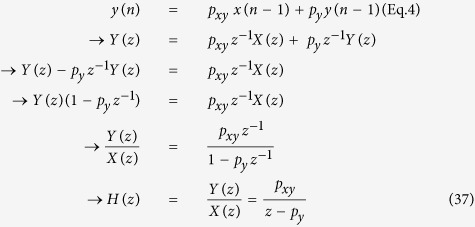


where 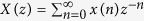, 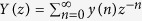, and *z* is a complex number. When performing the z-transform, the region of convergence, which is the set of points in the complex plane for which the z-transform summation converges, needs to be considered. *H*(*z*) is called the transfer function, which represents the system and transforms input *X*(*z*) into output *Y*(*z*). The frequency response of *H*(*z*) can be illustrated using Bode plot as shown in Fig. 8B (*p*_*xy*_ = 0.2, *p*_*y*_ = 0.9). The effect of extrinsic noise signals added to *x*_*protein*_ levels with cycle period shorter than 30 min (high-frequency noise) will be reduced in *y*_*protein*_ expression (Fig. 6B), indicating Eq. 4 is a low-pass filter that passes low-frequency *x*_*protein*_ signals while attenuating high-frequency *x*_*protein*_ signals. This way, cells can prevent unwanted noise signals from propagating from upstream genes to downstream genes. It is intriguing that this filtering mechanism is achieved by co-modulating protein production (*p*_*xy*_) and degradation (*p*_*y*_). Eq. 4 is also called an Infinite Impulse Response (IIR) filter in digital signal processing^1^. The response is “infinite” because there is feedback (*y*(n −1) → *y*(n)) in the filter.

The discrete Fourier transform (DFT) is a special form of the z-transform widely used in science and engineering (frequency analysis, fast convolution, image processing, etc.) because it can mathematically reveal periodicities (frequency components) in discrete-time data as well as the relative strengths of any frequency components^32^. For example, let us assume fluorescence intensity values of *x*_*protein*_ measured four times (the time-interval: 1 minute) are 2, 0, 4, and 7:


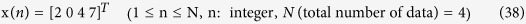


The DFT of the time series data *x*(n) can be computed using:


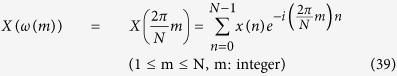


where *ω*(*m*) is angular frequency in radian per minute. 2π radian corresponds to one full cycle. For example, 4π radian per minute is equivalent to 2 cycles per minute. Since the total number of time-domain data N is 4, m can also take 4 different integer values, which generates 4 different *ω*(*m*) and *X*(*ω*(*m*)) values:

m = 0 (0 rad/min or 0 cycle/min):


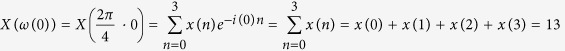


m = 1 (0.5π rad/min or 0.25 cycle/min):


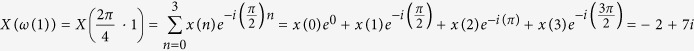


m = 2 (π rad/min or 0.5 cycle/min):


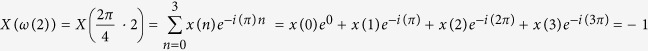


m = 3 (1.5π rad/min or 0.75 cycle/min):


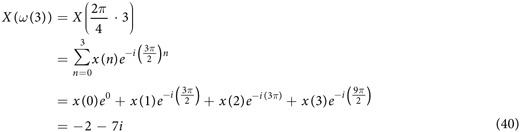


The magnitude of each complex number *X*(*ω*(*m*)) informs the relative strength of each frequency component, i.e., how much it contributes to the formation of *x*(n):


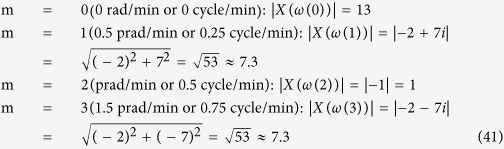


**Figure 9:**
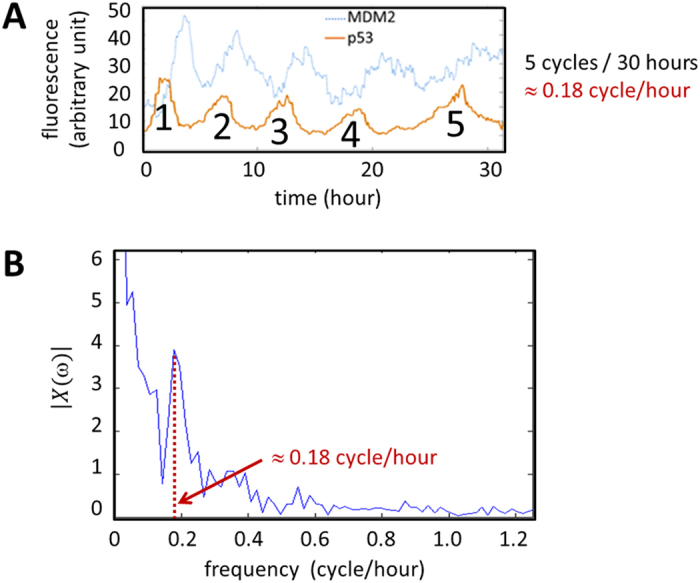
p53 oscillation. (**A**) There are about five cycles per 30 hours or 0.18 cycles per hour. (**B**) By letting *x*(n) represent the p53 protein levels, 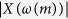 can be computed using Eq. 39. It is shown that the frequency component (0.18 cycle/hour) has high 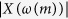 value represented by a tall peak, indicating that it is one of the prominent frequency components contributing to *x*(n).

The result of DFT is “mirrored” meaning that the values of 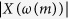 for 0 ≤ *ω*(*m*) ≤ π are equivalent to those of 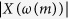 for π ≤ *ω*(*m*) ≤ 2π. In this respect, we can disregard the result for m = 3 (1.5π rad/min), which is equivalent to the result for m = 1 (0.5π rad/min). It was reported p53, one of the most studied genes in cancer biology, forms a negative feedback loop with MDM2 and p53 protein levels oscillate upon DNA damage by γ-irradiation^33^. Figure 9A shows such p53 oscillation. Note that there are about five cycles per 30 hours or 0.18 cycles per hour. By letting *x*(n) represent the p53 protein levels, 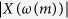 can be computed using Eq. 39 (Fig. 9B)^34^. It is shown that the frequency component 0.18 cycle/hour has high 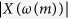 value represented by a tall peak, indicating that it is one of the prominent frequency components contributing to *x*(n).

### Autoregulation

**Figure 10:**
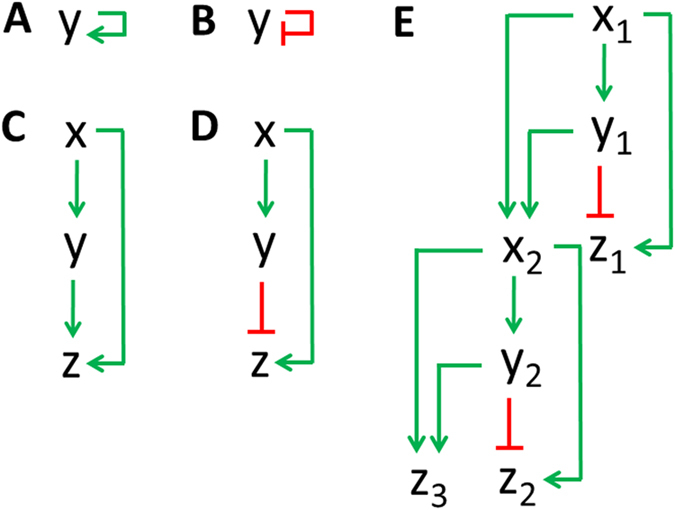
(**A**) Positive autoregulation (PAR). (**B**) Negative autoregulation (NAR). (**C**) Coherent type-1 feedforward loop (C1-FFL). (**D**) Incoherent type-1 feedforward loop (Ic1-FFL) (**E**) Interlocked feedforward loop.

Positive autoregulation (PAR) occurs when a gene promotes its own protein production (positive feedback, Fig. 10A). The equation for PAR can be obtained by adding an additional term, which represents positive autoregulation, to Eq. 4 (see learnsysbio.net **Module 10**^19^).


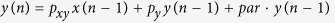


or


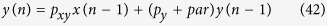


where *par* is the positive autoregulation parameter (*par* > 0). PAR increases the response time, the steady state value, and cell-cell variation in protein levels^8^. Negative autoregulation (NAR) occurs when a gene represses its own gene expression (negative feedback, Fig. 10B). The equation for NAR can be obtained by adding an extra term, which represents negative autoregulation, to Eq. 4 (see learnsysbio.net **Module 10**^19^).


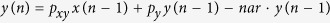


or


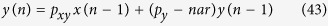


where *nar* is the negative autoregulation parameter (*nar* > 0). It is shown that NAR can decrease the response time and the steady state value, while reducing cell-cell variation in protein levels^8^.

### Feedforward loop

Coherent type-1 feedforward loop (C1-FFL) and incoherent type-1 feedforward loop (Ic1-FFL) are commonly found FFLs in biological systems^7^. It was reported that C1-FFL causes delay in gene expression while Ic1-FFL induces pulse-like transient gene expression. C1-FFL can be expressed using following equations (Fig. 10C):


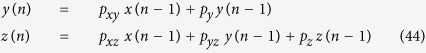


where three genes *x, y*, and *z* form C1-FFL, which induces delay in *z* (see learnsysbio.net **Module 11**^19^). Ic1-FFL can be expressed using following equations (Fig. 10D):


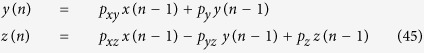


where three genes *x, y*, and *z* form Ic1-FFL, which induces pulse-like transient *z* gene expression (see learnsysbio.net **Module 12**^19^). C1-FFLs and Ic1-FFLs can be combined into more complex and larger gene networks. An example called interlocked FFL can be found in *Bacillus subtilis*, which generates sequential expression of multiple genes required for differentiation (see Fig. 10E and learnsysbio.net **Module 13**^19^,^35^).


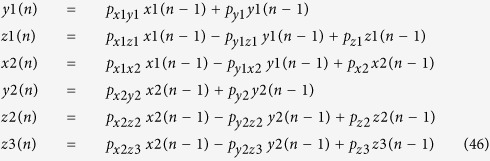


### Negative feedback control of protein levels

Gene networks are continuously affected by noise or fluctuations. Nevertheless, it is remarkable that they can robustly perform their functions in the presence of such disturbances. Feedback control theory informs us that feedback, a situation in which two (or more) dynamical sub-systems are connected in a way that their dynamics are coupled, can make a system resilient towards disturbances^36^. Feedback control theory can be used 1) to study the inherent robust feature of natural gene networks and 2) to manipulate the dynamics of genetically-modified gene networks with man-created control inputs. The main goal of the first approach is to gain insights, which may be experimentally validated using the second approach. For example, the author previously suggested that the p53-Mdm2 negative feedback gene network robustly achieves low levels of p53^11^. Since p53 protein may trigger apoptosis or “programmed cell death” when its levels are high, robustly maintaining them low using Mdm2 as a “negative feedback controller” becomes biologically significant. Although it is beyond the scope of this article, probing the uncertainty of gene network models, which may include various types of unmodeled dynamics (nonlinearity, etc.), is an important research topic^36^,^37^.

**Figure 11:**
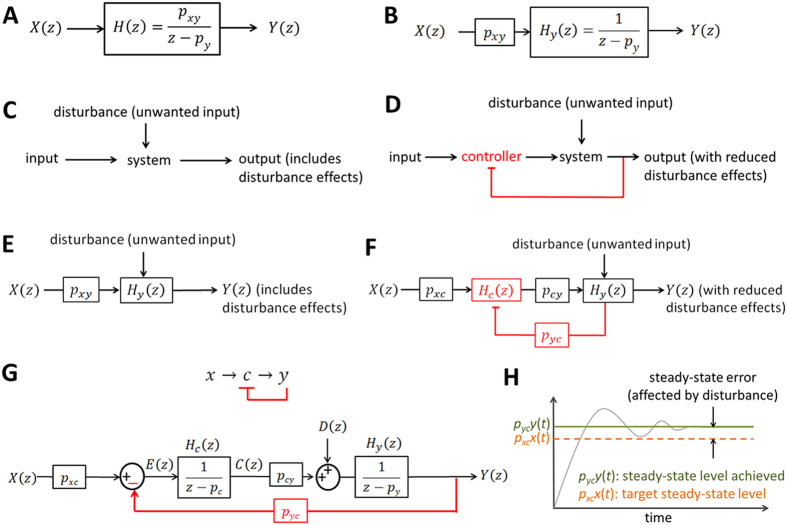
(**A**) Block diagram representation of Eq. 37. *X*(*z*) is the input, *Y*(*z*) is the output, and *H*(*z*) is the transfer or system function that processes the input to produce the output. (**B**) Since the system may have other inputs than *X*(*z*) we can redefine *H*(*z*) as *H*_*y*_(*z*) such that it includes only *p*_*y*_, which is independent of *X*(*z*). (**C**) When a system receives desired input signal in the presence of unwanted input or disturbance, the output is affected by both inputs. (**D**) The effect of such disturbance on the output can be reduced by adding a negative feedback loop and a controller to the system. (**E**,**F**) The same mechanism shown in *D* can be used to reduce of the disturbance effect on *y*_*protein*_ levels. (**G**) Block diagram representation of Eqs 47 and 48. (**H**) Steady-state error (the difference or error between target and actual steady-state levels as time goes to infinity) depends on disturbance.

Equation 37 can be represented by the block diagram shown in Fig. 11A. *X*(*z*) is the input, *Y*(*z*) is the output, and *H*(*z*) is the transfer or system function that processes the input to produce the output. Since the system may have other inputs than *X*(*z*) we can redefine *H*(*z*) as *H*_*y*_(*z*) such that it includes only *p*_*y*_, which is independent of *X*(*z*) (Fig. 11B). When designed properly, a negative feedback loop can reduce the impact of unwanted inputs or disturbances that influence the output of a system^38^. When a system receives desired input signal in the presence of unwanted input or disturbance (Fig. 11C), the output is affected by both inputs. The effect of such disturbance on the output can be reduced by adding a negative feedback loop and a controller to the system (Fig. 11D). The same mechanism can be used to reduce the disturbance effect on *y*_*protein*_ levels (Fig. 11E,F). A new controller gene “c” is added to the gene network, which forms a negative feedback loop with *y* (Fig. 11F). Discrete-time difference equations for this model can be expressed as:


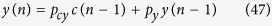



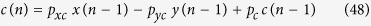


where all the parameter values are positive (see learnsysbio.net **Module 14**^19^). The negative sign for *y*(n − 1) term in Eq. 48 indicates that *y* is downregulating *c* (negative feedback).

### Steady-state error analysis

Steady-state error (the difference or error between target and actual steady-state levels as time goes to infinity) depends on disturbance, which can be analyzed mathematically^2^,^11^. Using the z-tranform, Eqs 47 and 48 can be represented by the block diagram shown in Fig. 11G. *E*(*z*) is the difference or error between *p*_*xc*_ *X*(*z*) and *p*_*yc*_ *Y*(*z*), which needs to be minimized, and distrubance is denoted as *D*(*z*). *E*(*z*) can be expressed as^11^:


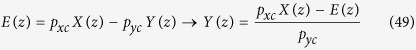


From the block diagram, *Y*(*z*) can also be written as:


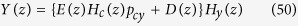


Substituting *Y*(*z*) in Eq. 50 by Eq. 49:


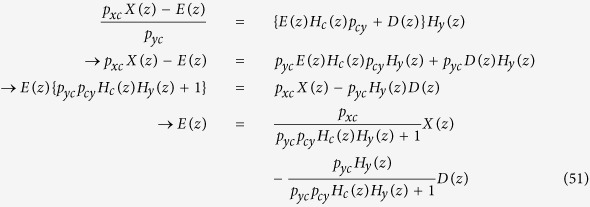


The second term in Eq. 51 represents the effect of *D*(*z*) on *E*(*z*) or the effect of the disturbance on the error, which needs to be minimized. This term can be denoted by *E*_*D*_(*z*) and its corresponding time domain discrete-time signal as *e*_*D*_(n). Using the final value theorem and assuming a step disturbance for *D*(z) (=z/(z − 1)), we can compute the steady-state error due to the disturbance as follows (Fig. 11H):


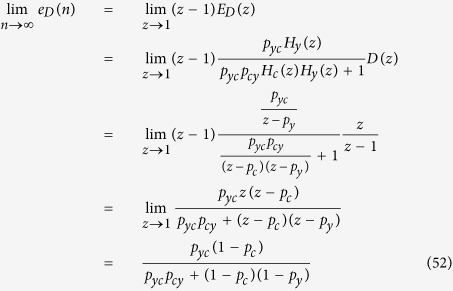


Interestingly, Eq. 52 provides insight that the steady-state error caused by the disturbance can be reduced when *p*_*cy*_ is increased or *p*_*y*_ is decreased^11^. In other words, how fast protein molecules are produced (*p*_*cy*_) and degraded (*p*_*y*_) can have an effect on the robustness of gene networks, although they may seem irrelevant.

### Stability analysis

Substituting *E*(*z*) in Eq. 50 by Eq. 49 and assuming no disturbance (*D*(*z*) = 0):


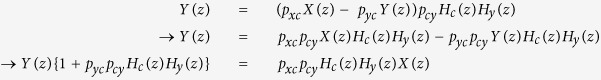



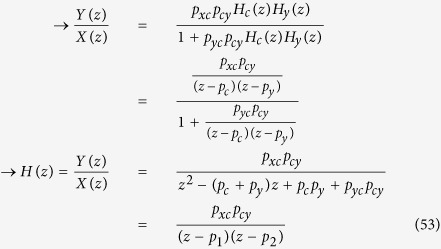


Complex numbers *p*_*1*_ and *p*_*2*_, which makes the denominator of *H*(*z*) zero, are called the poles of the transfer function. If the poles lie within the unit circle on the complex plane (the magnitude of each pole (complex number) is less than or equal to one), the system is stable and the output does not blow up^2^. From Eq. 11, a general form for *H*(*z*) can be derived:


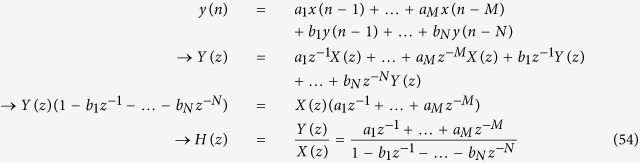


Again, the stability of this system can be analyzed by finding the poles and checking if they lie within the unit circle on the complex plane.

### Stability analysis using state-space representation

Equation 47 and 48 can be compactly represented by matrices and vectors:


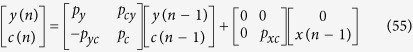


where 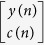 is the state vector, 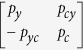 is the state matrix, 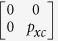
is the input matrix, and 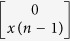 is the input vector. This is called state-space representation and the eigenvalues of the state matrix are equivalent to the pole values of transfer function shown in Eq. 53. If the magnitude of each eigenvalue is less than or equal to one, the system is stable.

### *In silico* digital control of protein levels

**Figure 12:**
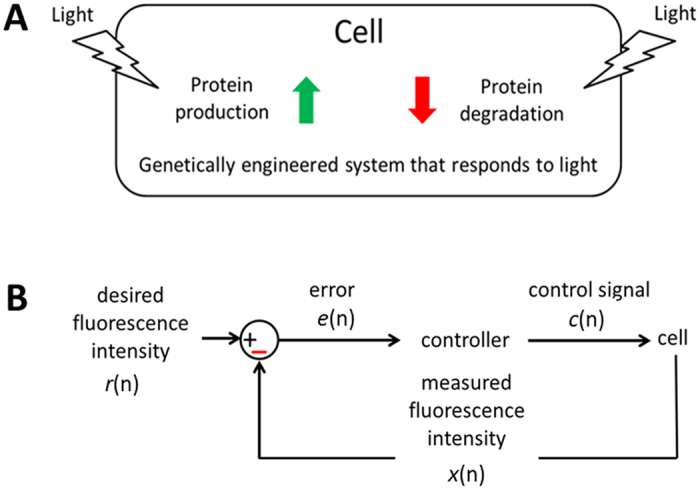
(**A**) Intracellular protein levels can be controlled using light by modulating protein production and degradation. Targeted time-varying protein levels in living cells are achieved using real-time fluorescent reporter imaging and *in silico* feedback control. (**B**) Desired or “reference” fluorescence intensity at time n is denoted as *r*(n). *e*(n) is the difference or “error” between *r*(n) and measured fluorescence intensity value *x*(n) at time n. Upon receiving error signal *e*(n), the controller uses this error and a control algorithm, such as the PID (proportional-integral-derivative) control, to compute control signal *c*(n), which is the intensity of light given to the cell.

As mentioned earlier, man-created control inputs can be used to manipulate the dynamics of genetically-modified gene networks, which may validate insights gained through mathematical modeling. One way of artificially modulating protein dynamics is using optogenetics, which was originally developed for modulating genetically modified light-sensitive ion channels in neurons^39^. For example, it was recently demonstrated that intracellular protein levels can be controlled using light by modulating protein production^40^,^41^ and degradation^42^ (Fig. 12A). Targeted time-varying protein levels in living cells are achieved using real-time fluorescent reporter image processing and *in silico* feedback control. Co-modulating protein production and degradation, harnessing the strengths of each mechanism, may generate protein dynamics previously shown in Fig. 4. Protein dynamics control virtually all cellular processes, including metabolism, growth, cell division, intercellular communication, etc., therefore, the capability of generating desired protein levels in individual cells can be immensely useful for unraveling the mechanism of these processes.

Figure 12B illustrates an *in silico* digital control scheme, which can be used to modulate protein levels using light. Desired or “reference” fluorescence intensity at time n is denoted as *r*(n). *e*(n) is the difference or “error” between *r*(n) and measured fluorescence intensity value *x*(n) at time n:


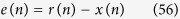


Upon receiving the error signal *e*(n), a control algorithm, such as the PID (proportional-integral-derivative) control, may generate a control signal *c*(n). It is the intensity of light given to the cell to minimize *e*(n). For example, *c*(n) generated by a PID controller can be expressed as:


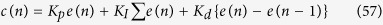


where *K*_*P*_, *K*_*I*_, and *K*_*D*_ are the controller parameters, which can be tuned for optimized performance and stability. Two types of *c*(n) signals can be used in parallel to co-modulate *x*(n) levels since protein production and degradation should rely on different colors of light to minimize cross-talk. Gene expression and relevant protein dynamics are slow processes, which take minutes and hours and not seconds, and the time interval between control input signals is typically greater than a minute^40^,^41^.

Real-time parameter estimation using adaptive filters described previously can be used for system identification, which may lead to better performance. For this, a simple mathematical model similar to Eq. 4 can be formulated as:


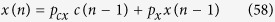


Note measured fluorescence intensity value *x*(n) at time n depends on two previous values at time n −1, *x*(n −1) and control signal *c*(n −1). Once *p*_*cx*_ and *p*_*x*_ are adaptively estimated, a set of PID controller parameters (*K*_*P*_, *K*_*I*_, and *K*_*D*_), which achieves desired performance and robustness, can be computed and updated in real time^43^. It is also possible to address model (parameter) uncertainty based on adaptively estimated parameter values and to design a controller using model predictive and robust control theory (robust adaptive control), although it is beyond the scope of this article^44^.

## Discussion

In this article, it was proposed digital signal processing and control provides useful tools for the study of gene networks. It was shown that discrete-time difference equations can be used to study complex dynamics of gene networks by incorporating time and frequency domain approaches. Most importantly, it was suggested that digital signal processing and control algorithms can be used not only to model, simulate, and analyze gene networks but also to interact with them in real time since the experimental environment is mostly digital today. By no means this article is comprehensive and many digital signal processing and control concepts and tools (e.g., band-pass filter, etc.), which can be useful for the study of gene networks, were not discussed and will be addressed in future studies.

## Methods

MATLAB (Mathworks, USA) and JavaScript programming language were used for simulation. The source code for online modules (learnsysbio.net) is available on GitHub^45^.

## Additional Information

**How to cite this article**: Shin, Y.-J. Digital Signal Processing and Control for the Study of Gene Networks. *Sci. Rep.* **6**, 24733; doi: 10.1038/srep24733 (2016).
